# PHYSICAL ACTIVITY ATTENUATES AGE DIFFERENCES IN CHANGE IN PERCEIVED PHYSICAL FATIGABILITY

**DOI:** 10.1093/geroni/igz038.3319

**Published:** 2019-11-08

**Authors:** Yujia (Susanna) Qiao, Theresa Gmelin, Robert M Boudreau, Stacy L Andersen, Stephanie Cosentino, Kaare Christensen, Mary K Wojczynski, Nancy W Glynn

**Affiliations:** 1 University of Pittsburgh, Pisstburgh, United States; 2 University of Pittsburgh. Department of Epidemiology, Pittsburgh, Pennsylvania, United States; 3 University of Pittsburgh, Pittsburgh, Pennsylvania, United States; 4 Boston University School of Medicine, Boston, Massachusetts, United States; 5 Columbia University, New York, New York, United States; 6 Danish Aging Research Center, University of Southern Denmark, Odense C, Denmark; 7 Department of Genetics, Washington University in St. Louis, Saint Louis, Missouri, United States; 8 University of Pittsburgh, Department of Epidemiology, Pittsburgh, Pennsylvania, United States

## Abstract

Lower physical activity is cross-sectionally associated with greater fatigability; whether such a relationship holds for longitudinal changes in fatigability is under-studied. We examined this question in offspring (≥60 years, range 60-93y, 99.7% white; 53.2% female) enrolled in the Long Life Family Study, a two-generation cohort enriched for exceptional longevity and their spousal controls. At Visit 2 (2014-2017), we measured self-reported physical activity (PA) with the Framingham Physical Activity Index (dichotomized by median value: less active <37 MET-hrs/wk and more active ≥37 MET-hrs/wk). Perceived physical fatigability was assessed using the Pittsburgh Fatigability Scale (PFS, 0-50) at Visit 2 and repeated during a follow-up contact 2.7±0.92 years later. We constructed a repeated-measures linear mixed-effect model to examine the effect of PA on longitudinal change in PFS by median age (younger <70y; older ≥70y) adjusted for family structure, field center, follow-up time, sex, and self-rated health. We found a strong dose-response relationship of PFS scores across the four age/PA groups (ptrend<0.001). Specifically, older/less active (N=310) participants had the highest annual PFS increases of 0.37 points/yr (p<0.001) while those older/more active (N=340) had annual increases of 0.17 points/yr (p=0.03). Younger/less active (N=371) participants had annual PFS increases of 0.09 points/yr (p=0.008); those younger/more active (N=341) had annual decreases (improvement) of 0.18 points/yr (p<0.001). Although annual PFS changes were modest, our findings indicate physical activity attenuated age differences in these trajectories. Physical activity is emerging as a potential target for intervention aimed at reducing fatigability - an important risk factor in the disability pathway.

